# Association between Cone-Beam Computed Tomography and Histological and Immunohistochemical Features in Periapical Lesions Correlated with Thickened Maxillary Sinus Mucosa

**DOI:** 10.3390/medicina57080840

**Published:** 2021-08-19

**Authors:** Alexandra Dumitrescu, Maria-Alexandra Martu, Alexandru Nemtoi, Ana Sirghe, Liliana Chelaru, Diana Tatarciuc, Ana-Maria Dumitrescu, Danisia Haba

**Affiliations:** 1 Department of Oral and Maxillofacial Surgery, “Grigore T. Popa” University of Medicine and Pharmacy, 16 Universitatii Str., 700115 Iasi, Romania; fo_ale@yahoo.com (A.D.); ana.petcu@umfiasi.ro (A.S.); danisia.haba@umfiasi.ro (D.H.); 2 Department of Periodontology, “Grigore T. Popa” University of Medicine and Pharmacy, 16 Universitatii Str., 700115 Iasi, Romania; 3 Department of Health and Human Development, “Stefan cel Mare” University of Suceava, 13 Universitatii Str., 720225 Suceava, Romania; alexandru.nemtoi@usm.ro; 4 Department of Morpho-Functional Sciences I, “Grigore T. Popa” University of Medicine and Pharmacy, 16 Universitatii Str., 700115 Iasi, Romania; liliana.chelaru@umfiasi.ro (L.C.); ana-maria-m-dumitrescu@d.umfiasi.ro (A.-M.D.); 5 Department of Internal Medicine, “Grigore T. Popa” University of Medicine and Pharmacy, 16 Universitatii Str., 700115 Iasi, Romania; diana.tatarciuc@gmail.com

**Keywords:** cone-beam computed tomography, immunohistochemistry, periapical granuloma, periapical cyst, odontogenic sinusitis, endo-perio lesions

## Abstract

*Background and Objectives*: Odontogenic sinusitis is a frequently underestimated pathology with fewer symptoms in patients with periapical lesions, periodontal disease, or iatrogenic foreign bodies in the maxillary sinus. The aim of our study was to determine the correlation between maxillary sinusitis and periapical lesions using cone-beam computed tomography (CBCT) imaging and histological and immunohistochemical investigations. *Materials and Methods:* A total of 1450 initial patients diagnosed with maxillary sinusitis in the Ear-Nose-Throat (ENT) Department, University of Medicine and Pharmacy “Grigore T. Popa” Iasi, Romania, were treated with anti-inflammatory drugs. Of these, 629 still had unresolved symptomatology and were later referred to the Dental Medicine departments for further investigations. Only 50 subjects with periapical lesions in the premolar/molar maxillary area were included in the present study. All the periapical lesions were observed on CBCT and classified using the Periapical Status Index (PSI) and the mean maxillary sinus mucosa thicknesses (MSMT). The enrolled patients underwent surgical procedures with the excision of periapical lesions. The excised samples were submitted to the histological and immunohistochemical investigations. *Results:* The 50 patients presented periapical lesions of their maxillary teeth in 328 dental units. There was a higher prevalence of periapical lesions in men than in women (chi-square test). We observed a significant difference between the mean MSMT of individuals with periapical lesions compared to those without (*p* < 0.01). Mean MSMT was 1.23 mm for teeth without periapical lesions and 3.95 mm for teeth with periapical lesions. The histopathological study identified 50% cases with periapical granulomas, 10% cases with periapical granulomas with cystic potential, and 40% cases as periapical cysts. Immunohistochemical stainings showed that CD4+ helper and CD8+ cytotoxic T lymphocytes, along with CD20+ B lymphocytes and CD68+ macrophages, were diffusely distributed in all periapical cysts and in some periapical granulomas, but CD79α+ plasma cells characterized especially periapical granulomas. *Conclusions:* The current study observed a significant correlation between CBCT maxillary mucosa thickness and type of periapical lesion. Chronic inflammatory lympho-histiocytic infiltrate predominates in periapical lesions, supporting the idea that lesion progression is determined by a humoral-type (CD20+ and CD79α+ B lymphocytes) but also by a cellular-type (CD4+ and CD8+ T lymphocyte population) immune mechanism.

## 1. Introduction

The maxillary sinus is part of the paranasal sinuses, situated in the middle of the face, is the largest amongst these and the first to develop [1]. At the same time, the maxillary sinus plays an important role in dentistry due to its anatomical position, which has an important clinical involvement. Recent research considers cone-beam computed tomography (CBCT) as a gold standard for studying anatomical variations of the maxillary sinus [2,3], odontogenic, or chronic rhino-sinusitis [4].

CBCT, although a fairly novel technique, has quickly become an important tool in the diagnosis of head and neck pathology, especially when discussing the maxillary bones and neighboring regions. One of the advantages that CBCT provides is that it can offer high resolution images of dental units and the surrounding tissues and a 3D image of the investigated area [5].

Sinus pathology can have different origins, and a substantial proportion is odontogenic, originating from different type of dental pathologies. Sinus pathology of dental origin is closely related to the spatial relationship of the posterior maxillary teeth, mainly molars and premolars, with the maxillary sinus. In addition to the anatomical relationship to the maxillary sinus, molars and premolars are the teeth that most frequently develop dental pathology, especially periapical [6]. Among the various dental conditions that lead to mucosal thickening and sinus pathology, the most frequent ones are apical periodontitis, incorrect or incomplete root canal treatments, severe periodontal disease, oroantral fistulas, extractions, and dental implants, especially in the context of a close anatomical rapport of maxillary teeth and sinus [7]. If the dental cause is not removed due to misdiagnosis, then the sinusitis becomes chronic and should be temporarily managed by antibiotic therapy [8]. However, this solution is not permanent and may lead to more severe complications, such as antibiotic resistance and possibly, in rare cases, life-threatening infections [9,10].

Periapical lesions represent an inflammatory process located around the dental apex that is caused by the presence of the bacterial infection inside the dental canals. The bacterial infiltrate spreads to the dental apex and beyond, causing morphological and histological alterations of the root and of the surrounding periapical tissue [11]. The most common periapical lesions are periapical granulomas and periapical cysts, differentiated by radiological examination, most often supplemented by histopathological examinations [12].

The aim of our study was to determine the correlation between the presence of maxillary sinusitis and various lesions of the apical region of maxillary teeth, using cone-beam computed tomography (CBCT) and assessing the degree of implication and modifications of the maxillary sinus mucosa. Furthermore, we performed pathological investigations, including immunohistochemical stainings, after the lesions were surgically removed in order to detect the type of inflammatory cells existing in the periapical lesions and to correlate the pathological features with the CBCT imaging.

## 2. Materials and Methods

### 2.1. Study Design

The present study was an observational study performed in the University of Medicine and Pharmacy “Grigore T. Popa” Iasi.

### 2.2. Study Group

The study was performed on 1450 initial subjects that presented in the otolaringology (ENT) department of the University of Medicine and Pharmacy “Grigore T. Popa” Iasi with a presumptive diagnosis of sinusitis of various etiologies, who were treated by otolaryngologist specialists with anti-inflammatory drugs. As the symptoms did not completely resolve, the patients were later referred to the Dental Faculty of The University of Medicine and Pharmacy “Grigore T. Popa” Iasi, Romania, between 1 November 2017 and 31 October 2020 for further investigations. Following dental clinical (vitality tests, affected tooth, endodontic status, percussion and palpation tests) and paraclinical (CBCT) diagnosis, we established that 629 patients had chronic periapical lesions in the maxillary premolars/molars that were associated with varying degrees of inflammation of the maxillary sinus mucosa.

Given that the literature shows significant variations in the incidence of chronic periapical lesions (10–86%) [13,14,15], in this study, we did not apply specific formulas for calculating the sample size. Thus, it was not possible to apply the specific formula for calculating the sample size based on incidence, $n\geq {\left(Z\left(1-\frac{\alpha }{2}\right)\right)}^{2}\cdot \frac{p\cdot \left(1-p\right)}{d^{2}}$ where Z = 1.96 for a 95% confidence interval, the value d corresponds to an estimation error of 5%, and *p* represents the known incidence in the literature. In the selection of patients, strictly the inclusion and exclusion criteria and the informed consent of the patients were taken into account.

Inclusion criteria for the study were the following: systemically healthy patients with a diagnosis of chronic periapical lesions in the maxillary premolars/molars established by an endodontic specialist, between ages 18 and 70, that agreed to participate in the study [11].

Exclusion criteria were the following: subjects with systemic diseases, pregnant or lactating women, subjects who had received periodontal or endodontic treatment in the last six months or antibiotic or anti-inflammatory treatment in the last three months, patients with implants, or patients with edentate maxilla. Patients under immunomodulatory, hormonal, anticoagulant therapy, or other type of medication or drug intake, as well as smokers were also excluded from the study [11].

Of the initial patients, only 50 were included in this study after the application of the inclusion and exclusion criteria and written consent to participate in the study. All patients were made aware to the purpose of the study and agreed and signed the informed consent prior to the start of the study. The methodology of the study complied with the rules set out in the Helsinki Declaration. The Ethics committee of The University of Medicine and Pharmacy “Grigore T. Popa” Iasi approved the study protocol (nr. 14.05.2015; 14 May 2015).

### 2.3. Cone-Beam Computed Tomography (CBCT) Examination

All 50 patients performed a maxillary CBCT, as indicated by dental specialists from the Dental Unit of “Grigore T. Popa” University of Medicine and Pharmacy Iasi. CBCT images were obtained from “Medimagis” Radiology Unit Iasi, Romania, using the Plamneca 3D Mid scanner (Plamneca, Helsinki, Finland) the field of view used was 10 × 200 mm, voxel size 200–400 µm, kV 80–85, mA 8–11, DAP 12.5 s–13.5 s. CBCT images were analyzed by a maxillo-facial radiologist who assessed the presence and severity of periapical lesions by using the Periapical Status Index (PSI), and identified the presence of sinusitis by measuring the thickness of the maxillary sinus mucosa.

All CBCT images were evaluated by one endodontic, one periodontal, one orthodontic, and one maxillofacial specialist. Evaluator calibration was done on a set of 30 previous CBCT images of patients not included in the study. All the evaluators assessed the CBCT images on two separate occasions, two weeks apart from the first evaluation, and the inter-evaluator reliability was 91%.

Using the PSI, the periapical status was evaluated as follows: (1) Normal periapical structures; (2) widening of the periodontal ligament, radiolucency with a major diameter of maximum 1 mm; (3) some mineral loss and changes in bone structure, major diameter of maximum 2 mm; (4) lesions with a well-defined radiolucent area, major diameter of 4 mm–8 mm; and (5) severe lesions with a tendency to expand, major diameter > 8 mm [16]. The thickness of the sinus mucosa was also evaluated by measurements made starting from the point of maximum thickness on the floor of the maxillary sinus over the apex of the maxillary teeth present. The thickening of the mucosa was present where the value was >1 mm. The maxillary sinus mucosa thickness (MSMT) was classified as follows: Class 1, normal (no mucosal thickness); Class 2, 0–2 mm; Class 3, 2–4 mm; Class 4, 4–10 mm; and Class 5, >10 mm [17].

### 2.4. Surgical, Histological, and Immunohistochemical Examination

After the imaging evaluation, in a period of time ranging from one week to eight weeks, the enrolled patients underwent surgical procedures, such as curettage after tooth extraction or apical resection, with the excision of pathological tissue from the apical region of the involved teeth, which were performed by an oral and maxillofacial surgeon.

Following surgical procedures, tissue samples of the apical region were preserved in 10% neutral buffered formalin, demineralized in case of bone lamellae presence into the sample, and then processed by the standard histopathological technique (inclusion of the sample into paraffin block, 4μ sectioning of the paraffin blocks using the microtome, and then staining of the histological sections with hematoxylin and eosin) [18].

Representative sections were submitted to the standard staining (Hematoxylin-Eosin staining) and then to immunohistochemical staining. In order to minimize the antigen distortion, the tissues were not exposed to temperatures above 60 °C during paraffin inclusion. For immunohistochemical staining, the paraffin sections were stretched on SuperFrostPlus slides that were initially incubated for 24 h at 370C for drying and firm adhesion. The IHC technique was performed in order to identify the antigens complementary to the following antibodies: anti-CD4, anti-CD8, anti-CD20, anti-CD68, and CD79α [18] (Table 1).

Immunohistochemical stainings for the identification of the antigens complementary to antibodies we used were performed using the BenchMark XT automatic staining system (Ventana Medical System, Inc., Tucson, AZ, USA) following protocols that required an initial standardization process of the method due to the fact that the antibodies we used are not manufactured by Ventana Medical System, Inc., Tucson, AZ, but by Novocastra [19,20].

During the working protocol, the slides were deparaffinized, and the endogenous peroxidase activity was blocked by incubation with a 3% H_2_O_2_ solution. Antigen unmasking was realized by the heat-unmasking technique in a slightly basic medium, pH = 9, using solution CC1, from Ventana (Ventana Medical System), which consists of a combination of EDTA and Boric Acid diluted in Tris buffer, a process that lasts between 30–60 min [19,20].

After treatment with normal goat serum 10% to block non-specific protein bonds, primary antibodies were applied by manual pipetting followed by incubation with conjugated horseradish multimer antibody (IgS; Ventana Medical Systems) [19,20].

The antigen-antibody reaction was visualized using diaminobenzidine as a chromogen (UltraView, Ventana Medical Systems), then the slides were stained with hematoxylin for counterstaining. The tissue was then dehydrated by immersing for 10 min in 2 successive baths of ethyl alcohol with increasing concentration, respectively, 90% and 100%, then followed by the air drying of sections for 10 min. The slides were clarified in two successive baths of high purity xylene solution for 10 and 20 min, respectively. Finally, the slides were mounted with a cover slip using a quick-mounting medium (Entellan) [20].

In order to monitor the correctness of the tissue processing, the staining procedures and the efficiency of the reagents, quality testing was performed by performing negative and positive external and internal controls. All slides were evaluated on a Leica Microsystem DM500LED microscope. The positive value of the label was assessed based on the presence of a brown precipitate whose specific membranous location represents the coupling site of the investigated molecule.

### 2.5. Statistical Evaluation

The data analyses were performed by using the SPSS 20.0 (SPSS Inc., Chicago, IL, USA). Numerical variables were presented as mean ± standard deviation. To compare the numerical series, the non-parametric Mann–Whitney U test was applied, specific to the distributions that show a significant difference between the dispersions. Categorial variables were presented as absolute frequency (*n*) and relative frequency (%). The association between categorial variables was assessed based on Pearson’s chi-square test results. Periapical status index and maxillary sinus mucosa thickness were considered primary outcome variables.

Patients included in the study were systemically healthy patients, which excluded the possibility of having a selection bias. We did not consider a multivariate logistic regression analysis necessary to highlight potential confounding variables (age, gender, etc.) that could significantly influence periapical status index (PSI) or maxillary sinus mucosal thickness (dependent variable).

For the selected patients, there were no independent variables (age, gender, etc.) that would significantly influence periapical status index (PSI) and maxillary sinus mucosal thickness. This was ensured by the inclusion and exclusion criteria applied.

Pearson correlation was applied to test the correlation between periapical status index and maxillary sinus mucosa thickness; evaluation of the link was made based on the correlation coefficient (r) and the significance level (*p*). For applied statistical tests, a value of the level of statistical significance less than 0.05 indicates statistical significance and accepts the alternative hypothesis.

## 3. Results

Of the initial 1450 patients, only 628 (43.3%) had periapical lesions in the premolar/molar maxillary area. Of these, only 50 subjects (7.96%) were included in the study. Demographic data of these patients are presented in Table 2.

**Figure 1:**
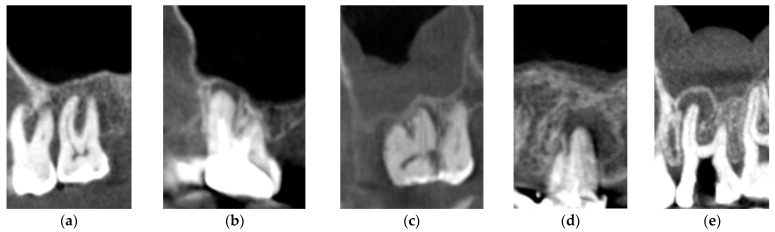
CBCT sagittal reconstructions showing the status of periapical region according to the five classes of PSI: (**a**). Class 1; (**b**) Class 2; (**c**) Class 3; (**d**) Class 4; (**e**) Class 5.

The CBCT images of 100 maxillary sinuses (Figure 1) and 328 teeth (88 first premolars, 81 s premolars, 85 first molars, and 74 s molars) of 50 patients were examined (Table 3). From the total of 100 maxillary sinuses examined, 92 presented different grades of thickness, which were measured on CBCT images (Figure 1). The results indicated a significant association between periapical lesions and patient gender (χ2 = 6.21, *p* = 0.002), which were more common in men (74%) (chi-square test).

Periapical lesions of the maxillary teeth were present in 328 teeth; depending on the location of the periapical lesions of the maxillary teeth, a higher frequency of cases with PSI = 5 was noticed in the case of the location of the right M1 (18.8%) and PSI = 4 on the right PM2 (18.9%) (Table 3).

The Mann–Whitney U test showed a significant difference between the mean MSMT adjacent to teeth with periapical lesions and those without (*p* < 0.01). Mean MSMT was 1.23 ± 0.6 mm for teeth without periapical lesions and 3.95 ± 0.8 mm for teeth with periapical lesions. In Figure 1a–e, we present conclusive images in this regard.

**Figure 2:**
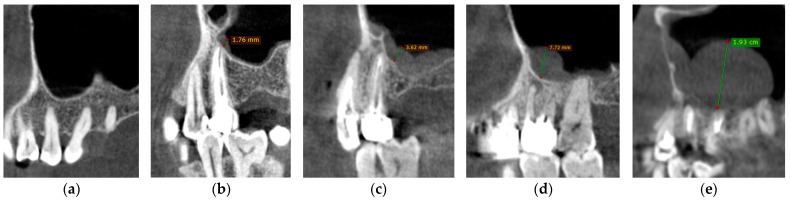
CBCT sagittal reconstructions showing the maxillary sinus mucosa thickness according to the five classes: (**a**) Class 1; (**b**) Class 2; (**c**) Class 3; (**d**) Class 4; (**e**) Class 5.

Among the examined sinuses, 7% had no mucosal thickness (Figure 2a), 13% presented uniform mucosal thickness <2 mm (maxillary sinus mucosa thickness/MSMT class 2) (Figure 2b), 20% mucosal thickness between 2–4 mm (MSMT 3) (Figure 2c), 27% with mucosal thickness between 4–10 mm (MSMT 4) (Figure 2d), and 33% with more than 10 mm, (Figure 2e) detected on the images in either the left or right maxillary sinus (Table 4). The mean mucosal thickness was 4.30 mm ± 2.23 (3.78 mm on the left side and 3.30 mm on the right side).

The prevalence of maxillary sinus mucosal thickness was 11.4% among the interval of 19–30 years, 21.4% among the 31–40 years, 33.2% among the 41–50 years, 39.3% among the patients aged between 51 to 60 years, and 33% among geriatric patients (>61 years).

**Figure 3:**
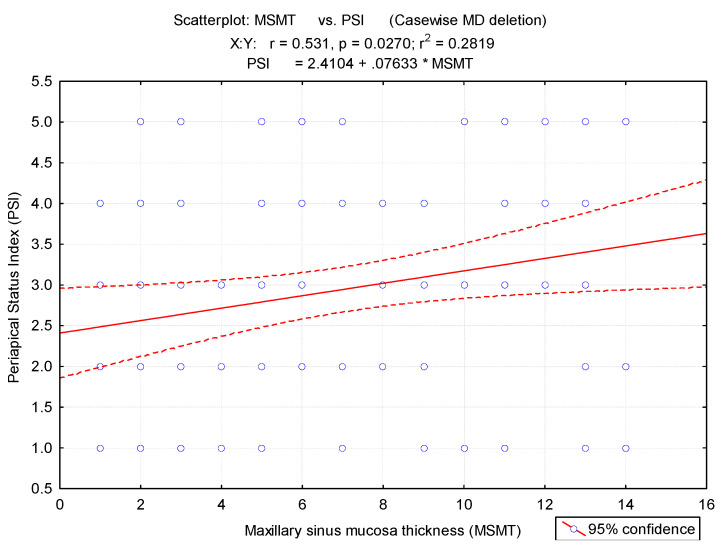
Correlation between the periapical status index (PSI) and maxillary sinus mucosa thickness. MS, maxillary sinus type.

There is a significant correlation between periapical lesions and mucosa thickness of maxillary sinus (r = 0.53, *p* < 0.001) (Figure 3). The possibility of maxillary sinus mucosa thickness increased dramatically as the degree of apical periodontitis increased.

The microscopical examination identified the histopathological type of periapical lesions. Thus, 15 (50%) cases were histologically diagnosed as periapical granulomas, 3 (10%) cases as periapical granulomas with cystic potential, and 12 (40%) cases as periapical cyst (Table 5). A significant association between the Periapical Status Index and the type of histopathological lesion was highlighted (*p* = 0.013). A significantly higher frequency of cases with PSI = 5 showing periapical cyst (75%) is noted (Table 5).

**Figure 4:**
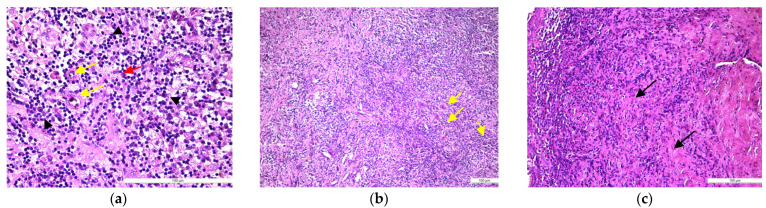
Histopathological features of periapical granuloma: (**a**) the early stage revealed a granulation (fibrovascular) tissue infiltrated by a heavy collection of chronic (mononuclear) inflammatory cells (black arrow head) but also few neutrophils (red arrow). There are numerous newly formed small blood vessels (yellow arrow) and thin, immature collagen in the background as well as numerous lymphocytes and plasma cells (HE staining, ×400); (**b**) the mature stage: granulation (fibrovascular) tissue infiltrated by a collection of chronic (mononuclear) inflammatory cells. There are numerous newly formed small blood vessels and fibrils of collagen in the background as well as numerous lymphocytes and plasma cells but also spindle shape fibroblasts, macrophages (histiocytes), and multinucleated giant cells (yellow arrow) (HE staining, ×100); (**c**) old stage: large sheets of inflammatory mononuclear cells included into heavy stromal fibrosis (black arrows) (HE staining, ×200).

**Figure 5:**
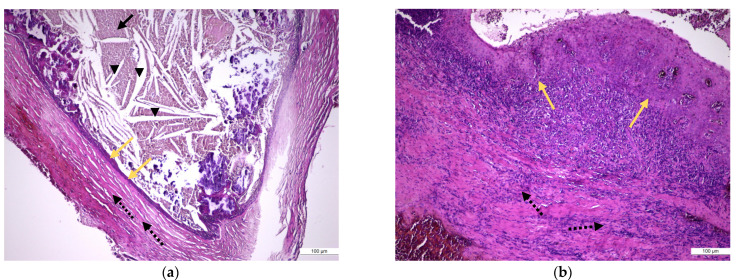
Histopathological features of periapical cyst: (**a**) low-power view of a large periapical cyst with a distended lumen that is lined by a non-keratinized stratified squamous epithelium (orange arrow) and is filled with an amorphous eosinophilic material (black arrow head), representing a coagulated fluid high in proteins but also with many cholesterol clefts. The cyst outer wall is thick and is made of fibrous conjunctive tissue (interrupted line arrow) containing a diffuse infiltration of inflammatory mononuclear cells (HE staining, ×10); (**b**) high-power view of the epithelial lining (orange arrow) but also of the outer fibrous wall that showed a heavy infiltrate with mononuclear inflammatory cells (interrupted line arrow) (HE staining, ×100).

In samples of periapical granuloma in their early stages, the standard staining with hematoxylin and eosin revealed a newly formed fibrovascular tissue (granulation tissue) made up of capillaries and thin collagen fibrils that was infiltrated with a variable proportion of mixed inflammatory cells, mainly lymphocytes, but also plasma cells, macrophages, and multinucleated giant cells (Figure 4a,b). In long-standing periapical granulomas, the number of newly formed blood vessels was reduced, but thick collagen fibers were identified, and the inflammatory infiltrate was very well represented (Figure 4c). The periapical cysts showed a lumen, an inner epithelial lining made up of a stratified squamous epithelium and a fibrous outer capsule, that was infiltrated by variable mixed inflammatory cells (Figure 5a,b).

**Figure 6:**
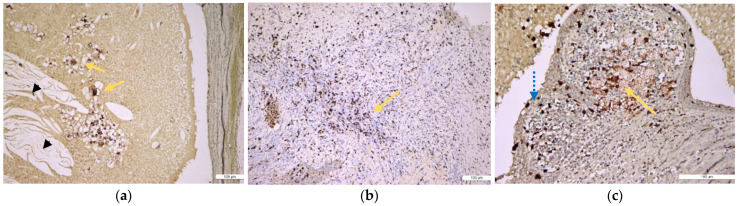
Immunohistochemical identification of the macrophages in the periapical cyst structure. (**a**) Periapical cyst lumen contained cholesterol crystals (black arrow head) and groups of CD68+ macrophages (orange arrow) (anti-CD68 antibody, ×100); (**b**) in the conjunctive fibrous tissue, which makes up the outer wall of the periapical cyst, there were numerous CD68+ macrophages (orange arrow) (anti-CD68 antibody, ×100); (**c**) periapical cyst wall with foamy CD68+ macrophages in the outer fibrous wall (orange arrow) but also into its epithelial lining (blue interrupted arrow) (anti-CD68 antibody, ×200).

**Figure 7:**
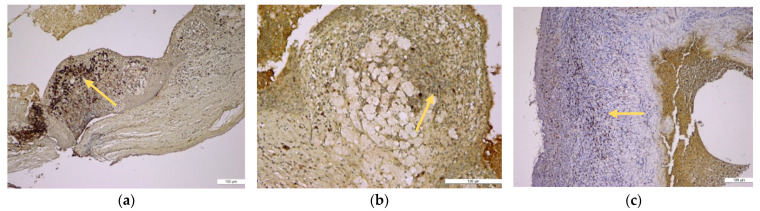
Immunohistochemical identification of the lymphocytes in the periapical cyst structure. (**a**) Numerous B lymphocytes, suggesting a follicular structure (orange arrow) located in the outer fibrous wall (anti-CD20 antibody, ×100); (**b**) numerous helper T lymphocytes in the outer fibrous wall (orange arrow) (anti-CD4 antibody, ×200); (**c**) diffuse inflammatory infiltrate made up of cytotoxic T lymphocytes that are located in the outer fibrous wall (orange arrow) (anti-CD8 antibody, ×100).

**Figure 8:**
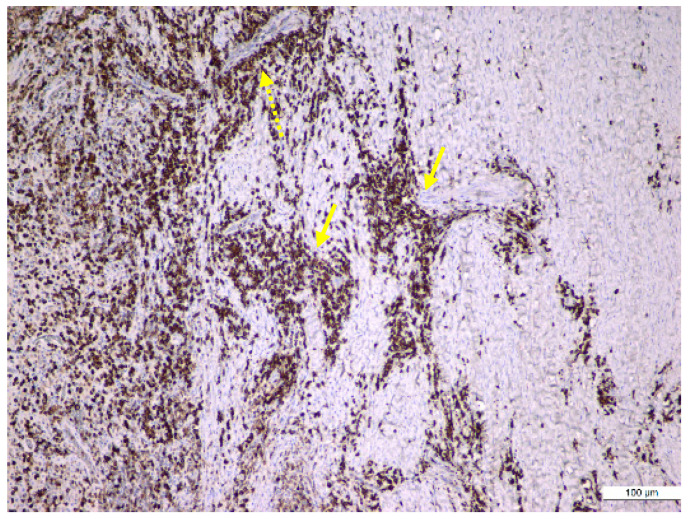
Immunohistochemical identification of the plasma cells in an old periapical granuloma: large sheets of plasma cells infiltrating the mature granulation tissue (yellow arrow), especially around the blood vessels (interrupted yellow arrow) (anti-CD79α antibody, ×100).

The results regarding the immunopositivity for CD4, CD8, CD20, CD68, and CD79α antibodies showed the following features: CD4+ helper T lymphocytes were distributed in all periapical cysts; CD8+ cytotoxic T lymphocytes were distributed in all periapical granulomas and cysts but were more numerous in the outer wall of the periapical cysts; CD20+ B lymphocytes were present in almost all periapical granulomas but also in the periapical cyst walls; CD68+ macrophages were present in all periapical cysts, mainly in the inner portion (subepithelial zone) of the outer fibrous wall; and in almost all periapical granulomas, CD79α+ B lymphocytes/plasma cells were present in an important number in the periapical granulomas (Figure 6, Figure 7 and Figure 8).

CD68+ macrophages were identified in the entire thickness of the wall but also in the content of the lumen (Figure 6). The distribution of the lymphocytic infiltrate in the lesions was usually diffuse for both types of lymphocytes, but CD4+ helper T lymphocytes showed a follicular pattern in some areas (Figure 7b).

## 4. Discussion

Sinus pathology of dental cause is a common affliction that oftentimes is under-diagnosed or misdiagnosed as having other causes; thus, treatment is ineffective.

In our study, we analyzed a total of initial 1450 patients of which 43.3% had chronic periapical lesions in the molar/premolar maxillary area. These data are similar to the prevalence reported in the literature, which estimates a range between 20% to 40%, with some authors reporting up to 60% [4,21]. Several other studies have reported an even wider range in prevalence, with rates from 10% up to 86% [13,14,15]. Maillet et al. reported that 98 of 135 MSMT cases were tooth related, presenting with changes in the maxillary sinus floor [22]. Periodontal disease, apical periodontitis, implant therapy, and tooth extraction are thought to increase the risk of maxillary sinus mucosal thickness [23,24,25]. Of these causes, marginal and apical periodontitis together constitute 83% of all dental causes [23].

Maxillary sinusitis is probably underdiagnosed in ENT and dental practice, as studies that use CBCT as a paraclinical aid in diagnosis report a higher prevalence of odontogenic sinusitis [26]. In the present study, we included only 50 patients with recurrent maxillary sinusitis on which we assessed the frequency of periapical lesions as well as their correlations with sinus involvement with the aid of CBCT examination and histological and immunohistochemical investigations. We only included 50 patients due to the strict inclusion and exclusion criteria, as we wanted to minimize the possibility of appearance of confounding factors, which could aggravate or mask any of the symptoms of sinusitis or influence the pathological results. CBCT is an imaging technique that has been used in oral and maxillofacial surgery, implant treatment planning, orthodontic evaluation, periodontal disease planning apical periodontitis assessment, and, in the last years, in different ENT pathologies diagnosis [27,28]. In our study, we used CBCT imaging in evaluating the preoperative periapical lesions of the maxillary teeth associated with varying degrees of the maxillary mucosa inflammation as an aid in determining the optimum surgical technique for removing the causative dental unit with as little as possible osseous tissue sacrifice and to avoid further sinus complications. Other studies have also stated its undeniable utility in pinpointing the possible etiological dental factor and evaluating the association between periapical lesions of maxillary teeth and the degree of involvement of the maxillary sinus [29,30].

In the present study, we evaluated the CBCT imaging characteristics of periapical lesions of the maxillary teeth and the relationship between maxillary sinus mucosa thickness and tooth pathology. There was significant difference in the MSMT between males and females, with a greater MSMT in males because of the presence of a larger number of periapical lesions. Our results were similar to those by Shanbhag et al., who observed a mucosal thickness >2 mm in 60.5% of analyzed patients and significant associations between mucosal thickening of more than 2 mm and sex (males), age (>60 years), and teeth with periapical lesions and periodontal disease (*p* < 0.027) [31].

Aksoy et al. found a mucosal thickening of more than 2 mm uni- or bilaterally in 58.5% of subjects, and the authors observed a significant correlation between mucosal thickening and age, gender, and missing teeth. The prevalence of mucosal thickening in maxillary sinuses with periapical lesions was 42.1 and without any periapical pathology was 53.6%, respectively [32].

A periapical lesion is an inflammatory process located at the tooth apex due to bacterial invasion and their toxins from the root beyond the apical stricture and into the surrounding periapical tissues. In our study, periapical granulomas were associated with less important modifications on the CBCT images compared with the periapical cyst, which showed a higher degree of inflammation of the sinus mucosa. The presence of periapical lesions resulted in an increase in MSMT, consistent with the results of other studies [33,34].

In our study, we established that mean MSMT and PSI positively correlated with the severity of periapical lesions and the anatomopathological diagnosis; thus, CBCT can be considered a reliable tool in establishing a correct diagnosis for patients with recurrent maxillary sinus inflammation. The microscopic analysis of all tissue samples used in our study showed a variable proportion of inflammatory cells, mainly lymphocytes, but also macrophages and plasma cells as well as fewer neutrophils, grouped as inflammatory infiltrates around newly formed vessels located in the cyst capsule. Furthermore, cholesterol crystals also have been seen in the lumen of the cysts, as Lin et al. identified in their study [35].

There is continuing controversy regarding the kind of inflammatory infiltrate that could be identified in periapical cysts. Some studies realized by Lin et al. and Marçal et al. found that mononuclear infiltrate was significantly more frequent than mixed infiltrate and that the latter was present in lesions with fistulae [35,36].

In our histological and immunohistochemical study, because the surgical interventions were made after the imaging evaluation in a period of time ranging from one week to eight weeks, we observed that periapical cysts removed after a short period of time contained in their wall few neutrophils among the predominant mononuclear inflammatory infiltrate, suggesting a subacute stage of the inflammation. In those lesions that were surgically removed after more than two weeks, only mononuclear cells, i.e., lymphocytes and plasmocytes but also multinucleated giant cells, were observed, which represent the histological appearance of the chronic stage of this lesion.

The presence of plasma cells in cyst walls suggest a local humoral immune reaction, as observed in another study [37].

Additionally, similar to other studies [38,39], we observed that cholesterol crystals move in the direction of the epithelium-lined cyst cavity since the outer collagenous capsule of the lesion is too challenging for the crystals to move through. Some researchers sustain the idea that the major source of cholesterol may be from locally dying inflammatory cells and a result of the disintegrating membranes of these cells in long-standing lesions. Accumulation of cholesterol crystals can prevent healing of apical periodontitis lesions, but accumulation of these biochemical structures does not seem to be associated with the development of periapical cysts, as the frequency of their presence is low in these lesions [40,41]. In our study, we occasionally observed that the periapical cysts are lined by columnar ciliated epithelium or muco-secretory cells, probably due to the migration of some cells from the maxillary sinus epithelium, especially in those lesions located in proximity to this anatomical structure.

A recent study compared the clinical characteristics, the sinus mucosa epithelial barrier integrity, and the morphological features of sinusitis of odontogenic but also rhinogenic origin. Biopsy specimens were harvested and analyzed immunhistochemically after nasal endoscopy. The formation of papillary mucosa folds and an enhancement of the barrier function were observed in odontogenic sinusitis patients as opposed to rhinogenic sinusitis and control subjects. Furthermore, in the sinusitis caused by dental factors, the inflammatory cells were predominantly lymphocytes and plasma cell phenotypes with a prevalence of T helper 17 cytokine profile [42].

Another study that evaluated the presence of plasma cells in apical periodontitis with regard to clinical and imaging characteristics observed no differences between periapical cysts and granulomas. Moreover, there was no statistically significant difference between CD68+ macrophages and CD138+ plasma cells when comparing the two groups [43].

In our study, quantification of the immunoreactivity of the inflammatory infiltrate revealed an important prevalence of CD20 + and CD79α + B lymphocytes despite the intense presence of CD8 + T lymphocytes, which have a cytotoxic effect. Similar to another study in the literature [44], our immunohistochemical reactions showed the presence of a dense, diffuse inflammatory infiltrate both at the level of the apical granuloma and in the wall of the periapical cyst. These diffuse inflammatory infiltrates are composed of both CD8 + and CD4 + T lymphocytes and CD20 + B lymphocytes as well as CD79α+ plasma cells and CD68 + macrophage cells, which suggests the presence of specific and non-specific defense mechanisms. Thus, it appears that B lymphocytes and T lymphocytes play an important role in the late phase of progression of periapical lesions, and CD8 + and CD4 + T lymphocytes probably exert regulatory or cytotoxic functions in the cellular immune response, which may lead to a stabilization of these lesions.

Healing after periapical lesions is of utmost importance, especially when considering the subsequent treatment plan that can involve implant placement or sinus lifting. The extent of the periapical lesion dictates the size of the osseous defect that results after dental extraction [45]. As such, the inflammatory infiltrate can play a major role in healing after dental extraction or apical resection [11,46]. Furthermore, in the context of additional grafting materials, the success of the surgical intervention can be put at risk; moreover, choice of implant length is affected, the clinician being forced to use a shorter option which could affect the long-term stability of the therapeutic result.

Limitations of our study consist of the small sample size of our final group and limitations in methodology; however, this was due to the strict inclusion and exclusion criteria. Future studies should be made on a larger sample size. In spite of this, our results emphasize the utility of CBCT in providing the practitioner a wide range of information regarding dental and maxillofacial pathology and its importance for patients with sinusitis symptoms and history of dental treatments, as they should have a holistic medical approach.

Even though few cases were analyzed, our study confirms the importance of investigating the sinus involvement of periapical lesions by dentists, otolaryngologists, and maxillofacial surgeons, as they should be evaluated by a medical team for the accurate management of patients.

The clinical implications of this study result from the fact that we observed a correlation between the periapical status index of dental lesions, maxillary sinus mucosa thicknesses, and the type and severity of inflammation in the periapical lesions, thus making CBCT a reliable, noninvasive diagnostic tool.

## 5. Conclusions

CBCT imaging shows an association between the presence of periapical lesions in the posterior area of the maxilla and thickening of the maxillary sinus mucosa. For patients with recurrent odontogenic symptoms, CBCT found that the chronic periapical cystic lesions were associated with an important thickness of maxillary sinus mucosa.

Periapical lesions are characterized by a dense, chronic inflammatory lympho-histiocytic infiltrate, supporting the idea that lesion progression is determined both by a humoral-type (CD20 + and CD79α + B lymphocytes) but also by a cellular-type (CD4 + and CD8 + T lymphocyte population) immune mechanism.

## Figures and Tables

**Table 1:** The antibodies used for immunohistochemical examinations.

Antibodies	Clone	Dilution	Distribution
CD4	Clone 4B12, code NCL-L-CD4-368, Novocastra, Leica Microsystem, UK	1:40	membranous
CD8	Clone 1A5, code NCL-L-CD8-295, Novocastra, Leica Microsystem, UK	1:80	membranous
CD20	Clone 1A5, code NCL-L-CD8-295, Novocastra, Leica Microsystem, UK	1:80	membranous
CD68	Clone 1A5, code NCL-L-CD8-295, Novocastra, Leica Microsystem, UK	1:80	membranous
CD79α	Clone 1A5, code NCL-L-CD8-295, Novocastra, Leica Microsystem, UK	1:80	membranous

**Table 2:** Demographical characteristics of studied patients.

Demographical Variables	
All Patients, *n*	50
Male, *n* (%)	29 (58)
Female, *n* (%)	21 (42)
Age, years, mean ± SD	39.8 ± 12.1

**Table 3:** Distribution of localization and class of periapical status index (PSI) on CBCT.

Localization	Periapical Status Index					
	1	2	3	4	5	Total
left PM1, *n* (%)	23 (46.9)	9 (18.4)	7 (14.3)	2 (4.1)	8 (16.3)	49
left PM2, *n* (%)	19 (43.2)	10 (22.7)	6 (13.6)	4 (9.1)	5 (11.4)	44
left M1, *n* (%)	10 (27)	15 (40.5)	5 (13.5))	3 (8.1)	4 (10.8)	37
left M2, *n* (%)	7 (18.4)	19 (50)	4 (10.5)	5 (13.2)	3 (7.9)	38
right PM1, *n* (%)	18 (46.2)	5 (12.8)	7 (17.9)	3 (7.7)	6 (15.4)	39
right PM2, *n* (%)	17 (45.9)	3 (8.1)	7 (18.9)	7 (18.9)	3 (8.1)	37
right M1, *n* (%)	9 (18.8)	19 (39.6)	10 (20.8)	1 (2.1)	9 (18.8)	48
right M2, *n* (%)	8 (22.2)	2 (5.6)	7 (19.4)	10 (27.8)	9 (25)	36

PSI, Periapical Status Index; PM, premolar; M, molar.

**Table 4:** Distribution of localization and class of maxillary sinus mucosa thickness (MSMT).

Localization	Maxillary Sinus Mucosal Thickness (Class)					
	Class 1	Class 2	Class 3	Class 4	Class 5	Total
left maxillary sinus mucosa, *n* (%)	6 (12)	4 (8)	8 (16)	14 (28)	18 (36)	50
right maxillary sinus mucosa, *n* (%)	2 (4)	5 (10)	4 (8%)	18 (36)	21 (42)	50
Pearson’s chi-square test: χ^2^ = 12.34, *p* = 0.0041						

MSMT, maxillary sinus mucosa thicknesses.

**Table 5:** The association between histological type of periapical lesions, CBCT features, and type of treatment.

	Histological Lesions			
	Periapical Granuloma	Periapical Granuloma with Cystic Potential	Periapical Cyst	*p*-Value
Cases, *n* (%)	15 (50)	3 (10)	12 (40)	
Periapical Status Index, 5/4/<3, *n* (%)	3/4/8(20/26.7/53.3)	1/1/1(33.3/33.3/33.3)	9/2/1(75/16.7/8.3)	0.013
MSMT, mm, (mean ± SD)	1.2 ± 0.4	2.6 ± 0.7	3.7 ± 0.8	0.002
**Treatment**				
dental extraction, *n* (%)	9 (60)	1 (33.3)	7 (58.3)	0.051
apical resection, *n* (%)	6 (40)	2 (66.7)	5 (41.7)	0.062

MSMT, mean maxillary sinus mucosa thicknesses.

## Data Availability

The data used to support the findings of this study are available from the corresponding author upon reasonable request.
